# *Operando* chemo-mechanical evolution in LiNi_0.8_Co_0.1_Mn_0.1_O_2_ cathodes

**DOI:** 10.1093/nsr/nwae254

**Published:** 2024-08-05

**Authors:** Yi Zhang, Shuaipeng Hao, Fei Pei, Xiangpeng Xiao, Chang Lu, Xing Lin, Zhe Li, Haijin Ji, Yue Shen, Lixia Yuan, Zhen Li, Yunhui Huang

**Affiliations:** State Key Laboratory of Material Processing and Die & Mould Technology, School of Materials Science and Engineering, Huazhong University of Science and Technology, Wuhan 430074, China; State Key Laboratory of Material Processing and Die & Mould Technology, School of Materials Science and Engineering, Huazhong University of Science and Technology, Wuhan 430074, China; State Key Laboratory of Material Processing and Die & Mould Technology, School of Materials Science and Engineering, Huazhong University of Science and Technology, Wuhan 430074, China; School of Optical and Electronic Information, National Engineering Laboratory for Next Generation Internet Access System, Wuhan National Laboratory for Optoelectronics, Huazhong University of Science and Technology, Wuhan 430074, China; Gatan Inc. & EDAX LLC., AMETEK Commercial Enterprise (Shanghai) Co., Ltd., Pilot Free Trade Zone, Shanghai 200131, China; State Key Laboratory of Material Processing and Die & Mould Technology, School of Materials Science and Engineering, Huazhong University of Science and Technology, Wuhan 430074, China; Department of Physics, The Chinese University of Hong Kong, Hong Kong 999077, China; State Key Laboratory of Material Processing and Die & Mould Technology, School of Materials Science and Engineering, Huazhong University of Science and Technology, Wuhan 430074, China; State Key Laboratory of Material Processing and Die & Mould Technology, School of Materials Science and Engineering, Huazhong University of Science and Technology, Wuhan 430074, China; State Key Laboratory of Material Processing and Die & Mould Technology, School of Materials Science and Engineering, Huazhong University of Science and Technology, Wuhan 430074, China; State Key Laboratory of Material Processing and Die & Mould Technology, School of Materials Science and Engineering, Huazhong University of Science and Technology, Wuhan 430074, China; State Key Laboratory of Material Processing and Die & Mould Technology, School of Materials Science and Engineering, Huazhong University of Science and Technology, Wuhan 430074, China

**Keywords:** Ni-rich layered oxide cathode, *in operando* stress monitoring, polycrystalline, chemo-mechanical evolution

## Abstract

Ni-rich LiNi*_x_*Co*_y_*Mn*_z_*O_2_ (NCM*xyz, x *+ *y *+ *z *= 1, *x *≥ 0.8) layered oxide materials are considered the main cathode materials for high-energy-density Li-ion batteries. However, the endless cracking of polycrystalline NCM materials caused by stress accelerates the loss of active materials and electrolyte decomposition, limiting the cycle life. Hence, understanding the chemo-mechanical evolution during (de)lithiation of NCM materials is crucial to performance improvement. In this work, an optical fiber with με resolution is designed to *in operando* detect the stress evolution of a polycrystalline LiNi_0.8_Co_0.1_Mn_0.1_O_2_ (P-NCM811) cathode during cycling. By integrating the sensor inside the cathode, the stress variation of P-NCM811 is completely transferred to the optical fiber. We find that the anisotropy of primary particles leads to the appearance of structural stress, inducing the formation of microcracks in polycrystalline particles, which is the main reason for capacity decay. The isotropy of primary particles reduces the structural stress of polycrystalline particles, eliminating the generation of microcracks. Accordingly, the P-NCM811 with an ordered arrangement structure delivered high electrochemical performance with capacity retention of 82% over 500 cycles. This work provides a brand-new perspective with regard to understanding the *operando* chemo-mechanical evolution of NCM materials during battery operation, and guides the design of electrode materials for rechargeable batteries.

## Teaser

This work highlights the grain orientation effect on the structural stress evolution of LiNi_0.8_Co_0.1_Mn_0.1_O_2_ cathodes via *operando* optical fiber monitoring, furthering our understanding of the *operando* chemo-mechanical evolution of NCM materials.

## INTRODUCTION

For widely used lithium-ion batteries (LIBs), enhancing their energy density and cycle life is crucial [1–5]. Benefitting from high specific capacity (>180 mAh g^−1^) and high working potential (3.8 V vs. Li/Li^+^), Ni-rich LiNi*_x_*Co*_y_*Mn*_z_*O_2_ (NCM*xyz, x *+ *y *+ *z *= 1, *x *≥ 0.8) layered oxide materials have been considered the main cathode candidates for high-energy-density LIBs and even solid-state Li metal batteries [6–10].

Conventional NCM layered oxide materials are typically synthesized by combining co-precipitation with high-temperature sintering, resulting in the formation of large spherical polycrystalline particles assembled from submicron-sized primary crystals, which are called polycrystalline NCM materials. Due to the primary-secondary architecture, the randomly oriented grain boundaries initiate anisotropic expansion and contraction during the (de)lithiation process, inducing stress concentration inside the particles [11–13]. Meanwhile, the lattice changes are aggravated after several cycles, leading to the intergranular crack generation of polycrystalline particles [14,15]. This opens new channels for electrolytes to infiltrate into polycrystalline particles and then increases the active surface area for parasitic reactions [16–18]. Hence, the intergranular cracking caused by stress concentration is recognized as the main degradation mechanism of polycrystalline NCM materials and how to detect and understand the stress evolution during (de)lithiation is crucial to performance improvement [19–21].

At present, many approaches have been proposed to evaluate the influence of stress on polycrystalline NCM materials (P-NCM). Through X-ray diffraction (XRD) and morphological observation, volume changes in crystal structure and particle cracks can be determined, confirming the mechanical failure of materials [22–24]. Further, finite element simulation can be performed to analyze the stress within the particles, figure out the stress distribution of materials and guide material optimization [25]. However, these approaches are usually based on *ex-situ* operations, or tend to rely on special cell configurations that are expensive and hardly used in normal operating conditions [26–28]. Few studies have been carried out to explore the chemo-mechanical evolution of P-NCM-based electrodes at με resolution under real working conditions.

In this work, an optical fiber sensor—a tiny, flexible and non-invasive *operando* technology—was applied to monitor the stress evolution of the NCM811 cathode electrodes. By designing the integration of the optical fiber sensor and electrode, the stress evolutions of different NCM811 electrodes have been successfully monitored at με resolution, reflecting a strong correspondence with the phase transition process of the materials. Combining the volume changes of crystal and morphological characteristics, the influence of chemical stress and structural stress on stress evolution was elucidated. Subsequently, the mechanical degradation mechanism of P-NCM811 has been revealed. With this understanding, we show that improving the arrangement structure of primary particles is the key to enhancing the performance of P-NCM811 materials. The P-NCM811 with an ordered grain orientation structure delivers a significantly enhanced cycling stability.

## RESULTS AND DISCUSSION

### The configuration for stress measurement

**Figure 1.**
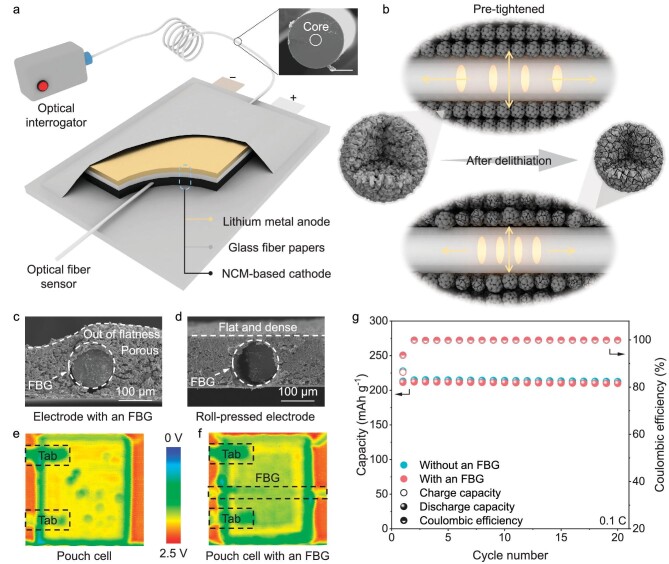
Experimental set-up for stress measurement. (a) Schematic illustration of the *in-operando* stress measurement system. Inset: cross-section SEM images of the optical fiber. The coating was removed before the cut for a flat cross-section. Scale bar, 50 μm. (b) Schematic illustration of the implanted FBG sensor and its surroundings. During cycling, the active particles suffered chemical reaction and volume changes, leading to changes of stress on the optical fiber. (c and d) Cross-section SEM images of the electrode with an FBG before (c) and after (d) roll-pressing. The loading of the electrode is ∼28 mg cm^−2^. (e and f) Ultrasonic transmission mappings of the normal pouch cell (e) and the pouch cell with an FBG (f). Although traces of the FBG sensor can be found by ultrasonic transmission mappings, the overall acoustic signal remains almost consistent. (g) Cycle performance of the pouch cell remained unaffected by the optical fiber.

A Fiber Bragg grating (FBG) sensor, a kind of optical fiber sensor, was employed to perform the stress measurement. Displayed in Fig. 1a and Fig. S1, the FBG sensor consisted of cladding and core, while having a grating at a specific location. The grating of the FBG sensor can be considered a reflector for a specific wavelength, which is called the Bragg wavelength. Owing to the elastic-optical effect and thermos-optical effect, the value of the Bragg wavelength was influenced by the changes in temperature and stress appearing in the surrounding environment of optical fiber. Hence, the stress and temperature signal can be monitored in real time via tracking the wavelength shifts [29]. Based on previous research, the shift of ${{\lambda }_B}$ can be defined as [30,31]:


(1) \begin{eqnarray*}
{\mathrm{\Delta }}{{\lambda }_B} &=& {{\lambda }_{B,0}}\left( {1 - {{p}_e}} \right)\varepsilon \nonumber\\
&=& \left( {1 - \frac{{n_{{\mathrm{eff}}}^2\left[ {{{p}_{12}} - \nu \left( {{{p}_{11}} + {{p}_{12}}} \right)} \right]}}{2}} \right)\varepsilon
\end{eqnarray*}

where ${{n}_{{\mathrm{eff}}}}$ is the effective refractive index, $\nu $ is the Poisson's ratio, and ${{p}_{11}}$ and ${{p}_{12}}$ represent the strain-optical coefficients (${{n}_{{\mathrm{eff}}}}$ = 1.45, ${{p}_{11}}$ = 0.113, ${{p}_{12}}$ = 0.252, $\nu$ = 0.17). Furthermore, Hooke's law ($\sigma = \varepsilon E$, E is Young's modulus of silica optical sensors equal to 69.9 GPa) is used to translate the strain into stress. The detailed calculation process is exhibited in Note S1.

**Figure 2.**
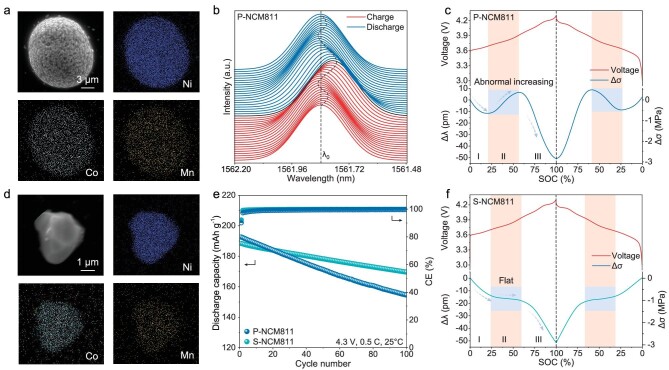
Stress evolution of NCM811 materials. (a) Scanning electron images of P-NCM811 materials with the corresponding elemental mapping of Ni, Co and Mn. (b) 2D stack-view of the reflected spectra given by the FBG sensor implanted into the P-NCM811 electrode during cycling. (c) Stress evolution of P-NCM materials, with corresponding voltage curves. (d) Scanning electron images of S-NCM811 materials with the corresponding elemental mapping of Ni, Co and Mn. (e) Cycling performance of P-NCM811 and S-NCM811 at 0.5 C (1 C = 180 mA g^−1^). (f) Stress evolution of P-NCM materials, with the corresponding voltage curves.

In order to monitor the stress evolution of NCM materials, the FBG sensor is implanted into a single-layered stacked Li||NCM pouch cell (Fig. 1a and b; Fig. S2). In view of the demand to increase the Ni content, polycrystalline LiNi_0.8_Co_0.1_Mn_0.1_O_2_ (P-NCM811) was chosen as the study object. According to our previous work [31], fiberglass papers were chosen as the separator, avoiding the crosstalk of the huge volume changes of lithium metal. Meanwhile, another FBG sensor was loosely placed on the side of the pouch cell, which can compensate for any temperature change in the surroundings. Subsequently, the combination of FBG sensor and NCM electrode was systematically studied. At first, following our previous work, the FBG sensor was placed on the surface of the electrode, as shown in Fig. S3. A shallow trench was formed on the P-NCM811 electrode in the region where the FBG sensor was. Meanwhile, the stress decreased during delithiation and increased during lithiation, which is consistent with the volume changes of NCM materials [32,33]. However, the slope of stress evolution remained basically constant, which cannot reflect the complex phase transition during cycling. Because the FBG sensor is fixed and pre-tightened, it fits tightly onto the surface of the electrode. During charge or discharge, the thickness of the electrode varied with the volume changes of active materials, which forced the FBG sensor to lengthen or shorten (Fig. S4). Hence, this integrated method mainly obtained the stress evolution at electrode level, with an absence of the stress information at the materials level. Hence, the FBG sensor was implanted into the electrode, as shown in Fig. 1c and Fig. S5a. After drying, the electrode was porous and uneven at the location of the FBG sensor. As shown in Fig. S6, the stress changes were not periodic and remained basically constant after several cycles. The porous structure of the electrode was considered to be the main reason for this result, which could balance out some volume changes [31]. Therefore, the prepared electrode was roll-pressed to be denser and flatter (Fig. 1d and Fig. S5b). Under the circumstances, the FBG sensor was mainly surrounded by active materials and there was also no room to balance the volume changes of active materials. Hence, there is any stress changes in materials during cycling, which can break the balance between the fiber and materials and force the stress state of the fiber to change (Fig. 1b). With this ingenious method, stress evolution at the material level has been successfully monitored and the related stress evolution is shown in Fig. 2c. Meanwhile, an ultrasonic imaging technique, developed by our group [34,35], was employed to investigate the effect of the FBG on electrolyte wetting. As shown in Fig. 1e and f, and Fig. S7, the peak-to-peak values of these two pouch cells exhibited little difference, indicating that the pouch cells were both wetting and not affected by the FBG sensor. Finally, the cycle performance of the pouch cell is displayed in Fig. 1g, indicating little influence of the FBG sensor on performance.

### Stress evolution of NCM811 materials

As shown in Fig. 2a, P-NCM811 was synthesized for stress measurement, and corresponding elemental mapping of Ni, Co and Mn demonstrated the uniform elemental distribution in the particles. XRD results indicate that P-NCM811 displays a typical layered hexagonal structure (Fig. S8). Following the exploratory method for stress monitoring, the reflected optical spectra taken in chronological order (from bottom to top) are displayed in Fig. 2b after smoothing, with the pristine Bragg wavelength denoted as ${{\lambda }_0}$. For all the reflected spectra, a single Bragg wavelength peak was seen obviously, whose changes represented the stress evolution of the P-NCM811 electrode. As shown in Fig. 2c, the stress evolution of the P-NCM811 electrode can be obtained by extracting all the Bragg wavelengths and removing the interference from the ambient temperature (shown in Fig. S9), which can correspond to the galvanostatic cycling curves and the corresponding voltage profile (Fig. S10a). Because the FBG was surrounded by materials and the electrode was roll-pressed to be dense, the stress caused by the volume changes of materials could be completely transferred to the FBG sensor (Fig. 1b). It is found that the stress (Δσ) exhibited a decreasing trend during charge while it increased during discharge, and Δσ had a minimum value of -2.84 MPa at a fully discharged status. Meanwhile, Δσ was also periodic and swung with voltage (Fig. S11), which also exhibited excellent reversibility. This phenomenon can be attributed to the chemical strain caused by the reversible chemical reaction of Li^+^ in P-NCM811 materials. With the increasing voltage, Li^+^ moved out of the lattice of P-NCM811, inducing the decreased volume of the unit cell. During discharge, Li^+^ returned to the lattice again and the crystal structure was restored. This reversible chemical reaction creates reversible chemical strain, which is also related to periodic stress evolution and cycling stability [36]. It is worth noting that the amplitude of change at a higher rate of 0.2C is smaller, which can be attributed to the capacity decay under a higher rate (Fig. S12). This further proves that chemical strain causes the generation of the structural stress.

However, it is interesting that Δσ can be divided into three parts and is not monotonous during charge or discharge, and Δσ briefly increased in region Ⅱ and had no relation with the rate of cycling (Fig. 2c and Fig. S12b). To decode the reasons behind the emergence of anomalous phenomena, single crystal NCM811 materials (S-NCM811) were synthesized for comparison [37–39]. The elemental mapping of Ni, Co and Mn demonstrated the uniform elemental distribution in the particles (Fig. 2d), and XRD results indicate that S-NCM811 also displays a typical layered hexagonal structure (Fig. S8). S-NCM811 exhibited an outstanding cycling stability with a capacity retention of 90% after 100 cycles, which was better than P-NCM811 with a capacity retention of 80% after 100 cycles (Fig. 2e). As shown in Figs S10b, S13 and 2f, Δσ of S-NCM811 materials has a similar tendency to that of P-NCM811 materials, and exhibited a minimum value of -2.91 MPa at full discharge. Moreover, Δσ was also reversible and periodic (Fig. S14), which was highly consistent with the P-NCM811 materials. In addition, Δσ also showed a much smaller variation at a higher rate of 0.2C (Fig. S15), indicating that it is also determined by chemical strain. However, the biggest difference between these two materials was that Δσ of S-NCM811 was monotonous during charge and discharge, which is shown more clearly in Fig. S16. More importantly, an obvious difference occurred at a voltage of 3.7–3.9 V, which should be decoded further.

**Figure 3.**
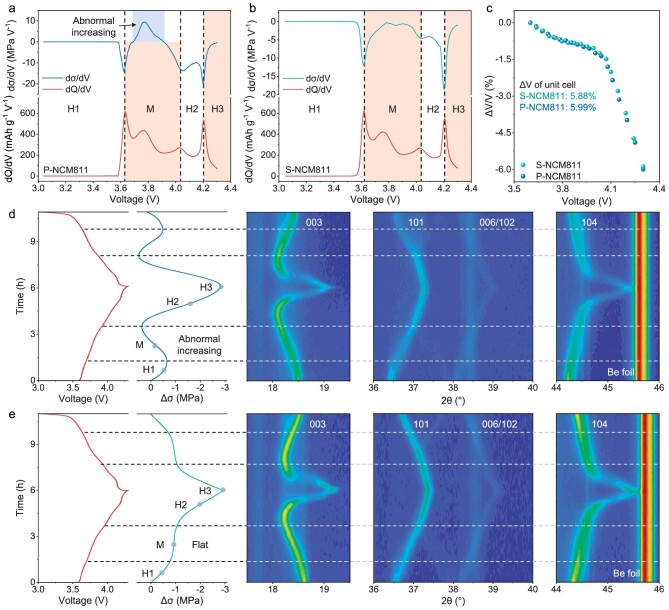
The crystal structural evolution and phase transition of NCM materials. (a and b) Voltage-resolved dσ/dV profile together with the dQ/dV plot of P-NCM811 (a) and S-NCM811 (b) materials, respectively. (c) Volume changes of the unit cell obtained from *in-situ* XRD. (d and e) *In-situ* XRD characterization of P-NCM811 (d) and S-NCM811 (e), with the corresponding voltage profiles and stress evolution. The evolution of the (003), (101), (006), (102) and (104) peaks are shown, respectively.

To better understand the chemo-mechanical evolution and phase transition of materials, the stress evolution is taken as a function of voltage, followed by the corresponding derivative of voltage. As shown in Fig. 3a, when the P-NCM811 was charged from 3 V to 4.3 V, the phase transformation process followed the order of the first hexagonal (H1), monolithic (M), the second hexagonal (H2), and the third hexagonal (H3), which can be significantly observed at the peak of dQ/dV. Meanwhile, the dσ/dV exhibited a peak near the voltage of phase transition (H1-M, M-H2, H2-H3), which can be attributed to the abrupt volume change of the unit cell caused by phase transition. This indicated that the phase transition process can also be detected by stress evolution. Moreover, an abnormal increase of Δσ was found to appear at the M phase. Likewise, except for the occurrence of the positive dσ/dV, the S-NCM811 materials stayed almost the same as P-NCM811, indicating the occurrence of similar reversible chemical reactions (Fig. 3b). In addition, the stress evolution of P-NCM811 and S-NCM811 at high voltage (3–4.6 V) has also been monitored (Fig. S17). Obviously, the trend of stress evolution has not changed, and the difference caused by the wide voltage range of 3–4.6 V is that the stress of these two materials is slightly greater than that of the 3–4.3 V voltage range. As the voltage range expanded, more lithium ions were involved in the reversible chemical reaction, leading to a further increase in chemical stress.

Furthermore, *in-situ* XRD was employed to analyze the crystal structural evolution of materials during cycling (Fig. 3d and e). When the P-NCM811 was charged from OCV to 4.1 V, the (003) peak underwent a continuous shift to lower 2θ, corresponding to the expansion of interslab distance along the c-axis [40]. This is attributed to the columbic repulsion between the adjacent layers within the delithiated unit cell induced by H1-M-H2 phase transformation [41]. Subsequently, upon further charging to 4.3 V, the H2-H3 phase transition led to a drastic contraction of the c-axis, resulting in a sharp shift of the (003) peak to high 2*θ*. The other peaks, such as (101), (006), (102) and (104), exhibited a monotonous shift to higher 2θ, attributed to the contraction of the a-b plane with a decrease in the ionic radius of transition metal ions (the charging process over the transition metal valence state increases) [22]. The phenomenon of S-NCM811 materials was similar with P-NCM811 materials, indicating that the structural evolution was almost highly consistent. Further, according to the XRD curve, ΔV of the unit cell was figured out and showed a monotonous decrease during delithiation, proving that the chemical strain caused by chemical reaction is the main cause of stress evolution (Fig. 3c). The volume change of the unit cell for S-NCM811 was 5.88%, which was smaller than that of P-NCM811 (5.99%), indicating better structural reversibility and excellent cycle performance for S-NCM811. At this point, it is easily deduced that chemical strain is not the cause of the difference in stress between them. Notably, the trend of stress evolution in P-NCM811 was more similar to the trend of (003) peak shift, while the trend of stress evolution in S-NCM811 was more similar to the trend of (101) and (104) peak shift. The shift of peak often depends on the variation of lattice parameters, which shall be discussed subsequently.

### Mechanistic analysis for the stress anomalies

**Figure 4.**
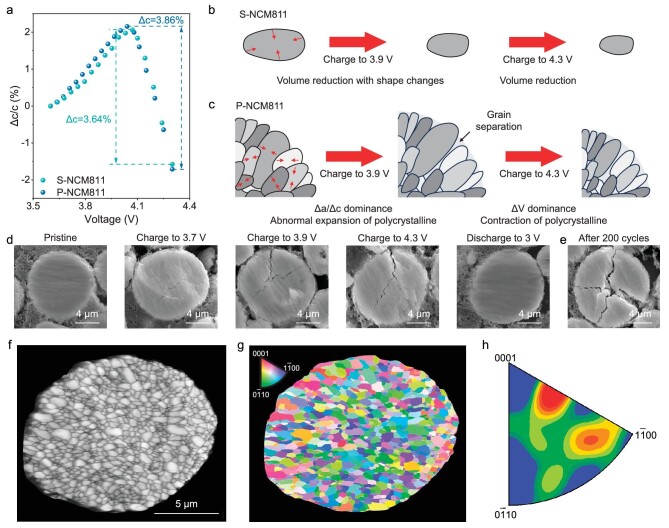
Mechanistic analysis for the stress anomalies. (a) The variation of c-axis parameter for P-NCM811 and S-NCM811 during the charging process. (b) Schematic diagram of chemical stress-dependent S-NCM811 particles during charge. (c) Schematic diagram of chemical stress-dependent S-NCM811 particles during charge. (d–e) Cross-section SEM images of P-NCM811 particles during the fifth cycle (d) and after 200 cycles (e). (f) SEM images of cross-sections of P-NCM811 particles. The polycrystalline particles consist of many primary particles and the grain boundary is obvious. (g and h) EBSD images and crystal plane distribution of P-NCM811 particles.

As per the *in-situ* XRD measurement, the variation of lattice parameters a and c is shown in Fig. 4a and Fig. S18. The change of lattice parameter a (Δa) and c (Δa) for P-NCM811 and S-NCM811 is similar, except that the variation of P-NCM811 is higher, confirming that the reversibility of chemical reactions in S-NCM811 is higher. Meanwhile, Δa is found to be decreasing persistently and Δc increases first and then decreases, indicating that the shape of the crystal lattice changed slightly during cycling. Although the volume of the crystal has been reduced, the shape has changed slightly during delithiation. Hence, it is necessary to examine the morphological structure of these two materials and the stress mode of FBG sensors. At first, the FBG sensor was surrounded by active materials and the electrode was roll-pressed to be dense, resulting in the volume changes of materials being almost transmitted to the FBG sensors (Fig. 1b). For the S-NCM811 electrode, the FBG sensor has been fully influenced by a single crystal. Although the shape of the crystal has changed, the volume of the crystal is always reduced during delithiation, resulting in less stress on the FBG sensor (Fig. 4b). However, the situation changed for polycrystalline, which consists of many primary particles. During delithiation, the shape of each primary particle in the spherical polycrystalline particles changes and their volume decreases. The combined effect of these two factors on the volume changes of polycrystalline is debatable. Further, the morphological structure of P-NCM811 was characterized. As shown in Fig. 4d, it is found that the polycrystalline particles cracked during charging and recovered after discharging, indicating the appearance of interaction between the previously arranged primary particles’ grain boundaries [3,42–44]. This strong interaction leads to the formation of microcracks.

To explore the reason for the interaction, the electron back scatter diffraction (EBSD) results are shown in Fig. 4f–h. Red, blue and green represent (0001), ($1\bar{1}00$) and ($0\bar{1}10$) crystal faces, respectively. With the increase in chromatic aberration, the crystal plane angle becomes larger. The primary particles of P-NCM811 exhibit irregular shapes, and the crystal planes’ orientation between adjacent grains is quite different, indicating the anisotropy of primary particles [45]. According to the crystal plane distribution (Fig. 4h), it can be seen that the distribution of P-NCM811 particles in three crystal planes is relatively dispersive, with a lack of preference orientation, which indicates that there is no obvious rule for the distribution of grains in P-NCM811 particles. Hence, the anisotropy of the primary particles and the shape change of the single crystal during delithiation lead to the formation of cracks, which can also influence the volume change of polycrystalline particles and increase the volume of individual polycrystalline particles. With the combination of these two factors, the reason for stress evolution can be decoded. As shown in Fig. 2c, the decrease of crystal volume is more than the increase of cracks in region Ⅰ, which is the main factor affecting the stress, so the stress shows a decreasing trend. Subsequently, with the further increase of c-axis, the shape changes of the single crystal dominate and result in the increased volume of polycrystalline particles, which leads to the abnormal increasing stress. In region Ⅲ, due to the rapid reduction of the c-axis, the volume change retook the dominant position, resulting in a rapid reduction in stress. The overall process is shown in Fig. 4c. However, the process had changed for S-NCM811. Although each particle exhibited different orientation according to the results of EBSD (Fig. S19), there is no crack inside the particle and no strong interaction between the particles, which facilitated the monotonicity of stress evolution during charge/discharge. Hence, the abnormal increasing of stress evolution is highly related to the formation of cracks within polycrystalline particles, which also determines the cycling stability of materials. As shown in Fig. 4e, a large number of cracks were generated after 200 cycles, which resulted in the capacity fade. This is the effect of the structure of polycrystalline particles on stress evolution, which can be called structural stress. Therefore, the elimination of cracks, which means the elimination of structural stress during cycling, is the key to improving performance.

### Optimize stress for high performance based on ordered arrangement structure

**Figure 5.**
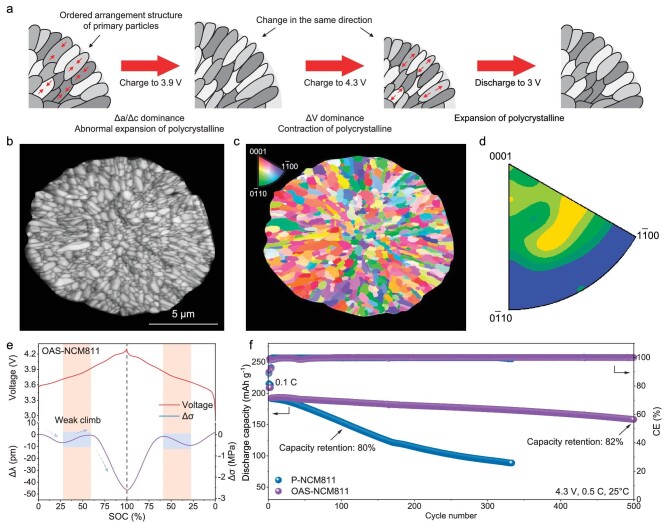
Optimize stress for high performance based on ordered arrangement structure. (a) Schematic diagram of chemical stress-dependent S-NCM811 particles during cycling. (b) SEM images of cross-sections of OAS-NCM811 particles. (c and d) EBSD images and crystal plane distribution of OAS-NCM811 particles. (e) Stress evolution of OAS-NCM811 materials, with the corresponding voltage profile. (f) Cycling performance of P-NCM811 and OAS-NCM811 at 0.5C. The loading of electrodes is ∼3 mg cm^−2^.

Based on the above discussion, structural stress is an expression of crack formation within stress evolution. Hence, the factors that resulted in an abnormal increase in stress are as follows: (i) polycrystalline particles being composed of anisotropic primary particles, and (ii) the shape changes of primary particles as the c-axis increases and then decreases. Accordingly, there are three ways to relieve the structural stress. At first, the polycrystalline particles are completely transformed into single crystal particles, which has been discussed previously. The stress evolution of S-NCM811 remained monotonous during charge and discharge and the cross-section of particles also remained flat after 200 cycles (Fig. S20), which resulted in better cycling performance. Secondly, the c-axis is transformed into a monotonic reduction during charge. This keeps the crystal structure from changing shape and the stress evolution is dominated by the volume change of the crystal. Finally, the anisotropy of the primary particles is reduced so that they have an ordered arrangement structure. In this case, the primary particles change volume in the same direction, reducing the likelihood of cracks (Fig. 5a).

Accordingly, element doping was adopted to modify the P-NCM811 materials, with the introduction of Gd elements. As show in Fig. S21, the Gd-doped P-NCM811 was synthesized, and corresponding elemental mapping of Ni, Co, Mn and Gd demonstrated the uniform elemental distribution in the particles. Further, the cross-section of a Gd-doped P-NCM811 particle was shown in Fig. 5b, exhibiting an ordered arrangement structure (called OAS-NCM811). In addition, the result of EBSD (Fig. 5c and d) shows that the crystal plane orientations of adjacent primary particles from the outside to the inside are similar, and the crystal plane mainly concentrated on (0001). Hence, these results all prove that the primary particles in OAS-NCM811 particles are isotropic. Subsequently, stress monitoring was carried out for OAS-NCM811 materials during cycling (Fig. 5e and Figs S22–S24). Although the structural stress still appeared in region Ⅱ, the rise amplitude is greatly reduced compared with P-NCM811, showing that the influence of structural stress on stress evolution becomes weaker in region Ⅱ. In addition, according to *in-situ* XRD measurements (Fig. S25), the change of c-axis is also lower than that of P-NCM811, indicating structural stress reduction due to shape changes. The above results indicate that when the grain growth directions of primary particles in secondary particles converge, the stresses generated by the deformation of primary particles during charging and discharging can be canceled out with the radial direction, which reduces the generation of particle microcracks. Finally, Li||OAS-NCM811 was assembled to demonstrate the electrochemical performance. As shown in Fig. 5f, OAS-NCM811 materials deliver a high-capacity retention of 82% after 500 cycles at 0.5C, which is significantly higher than that of P-NCM811 materials (80% capacity retention after 100 cycles). Meanwhile, compared to P-NCM811 particles, the microcracks barely generated in the particles of OAS-NCM811 after 200 cycles (Fig. S26).

## CONCLUSION

In summary, optical fiber sensors were buried inside various NCM-based electrodes to *in operando* investigate the chemo-mechanical evolution of NCM811 materials during cycling, and the stress evolution at material level was successfully monitored. It is found that stress evolution can be strongly related to the chemical reaction and phase transition of materials, which further reveals the chemo-mechanical events of NCM materials. By comparing the stress evolution of S-NCM811 and P-NCM811, an abnormal stress increase is observed during delithiation of P-NCM811. Combining crystallography and morphology, it can be concluded that stress evolution is affected by both chemical stress and structural stress. The abnormal stress increase of P-NCM811 is related to the structural stress, which was induced by the anisotropy of primary particles. To eliminate the effect of structural stress in order to achieve high performance, Gd-doped NCM811 materials were synthesized to construct polycrystalline particles with an ordered arrangement structure. The isotropy of primary particles in polycrystalline particles reduces the generation of cracks. As a result, OAS-NCM811 materials deliver a high-capacity retention of 82% after 500 cycles at 0.5C. This work highlights the adverse effect of structural stress on cycling performance, providing a new understanding of the chemo-mechanical evolution of polycrystalline Ni-rich layered oxide cathodes.

## METHODS

### Materials synthesis

Ni_0.8_Co_0.1_Mn_0.1_(OH)_2_ and LiOH·H_2_O were mixed with a molar ratio of 1 : 1.05 and calcined at 850°C (12 h) to prepare the single-crystal LiNi_0.8_Co_0.1_Mn_0.1_O_2_ cathode materials (S-NCM811). Ni_0.8_Co_0.1_Mn_0.1_(OH)_2_ and LiOH·H_2_O were mixed with a molar ratio of 1 : 1.05 and calcined at 500°C (3 h) and 750°C (12 h) to prepare the polycrystalline LiNi_0.8_Co_0.1_Mn_0.1_O_2_ cathode materials (P-NCM811). Ni_0.8_Co_0.1_Mn_0.1_(OH)_2_, LiOH·H_2_O and Gd_2_C_2_O_4_·10H_2_O were mixed with a molar ratio of 1 : 1.05 : 0.005 and calcined at 500°C (3 h) and 750°C (12 h) to prepare the Gd-doped polycrystalline LiNi_0.8_Co_0.1_Mn_0.1_O_2_ cathode materials (OAS-NCM811).

### Cell assembly

Each active material was mixed with super P and poly (vinylidene fluoride), with a mass ratio of 93 : 3.5 : 3.5, in N-methyl pyrrolidone to form a slurry. The slurry was coated onto Al foil and dried in a vacuum oven at 70°C for 12 h. For the electrode with an FBG, the FBG was placed on the Al foil with both ends fixed in advance and the other steps were the same as the common electrode. Subsequently, the pouch cells were assembled to research the stress evolution of an NCM electrode. The prepared electrodes and the fresh lithium foils (thickness of 100 μm) were cut into appropriate sizes. The NCM electrodes and lithium foil were stacked up and down, separated by fiberglass papers. The electrolyte was purchased from Duo-duo Chem Co., Ltd., and contained 1 M LiPF_6_ in ethylene carbonate and diethyl carbonate (v : v = 1 : 1) with 5 wt% fluoroethylene carbonate. The pouch cell was filled with 4 mL electrolyte and rested for 12 h, then was ready to test. The loading of the electrode for pouch cells is ∼28 mg cm^−2^. In addition, Li||NCM811 coin cells were assembled for performance test. The loading of the electrode for coin cells is ∼3 mg cm^−2^. All the above operations were performed in an argon-filled glove box (H_2_O < 0.1 ppm, O_2_ < 0.1 ppm).

### Electrochemical tests

Cycling performances of the pouch cells were measured on a Neware electrochemical testing system (CT-9040-5V5A-G4T) in an oven at constant temperature. The cells were cycled between 3 V and 4.3 V, and the detailed rate has been indicated.

### Materials characterization

The morphologies of materials were characterized by a field-emission scanning electron microscope (SEM, FEI Nova NanoSEM450). The samples were attached to the sample table with conductive adhesive and transferred to the sample chamber of the SEM. To obtain the cross sections for the SEM and EBSD, the particles were cut by focused ion beam. The EBSD characterization was conducted on a JEOL field-emission SEM (JSM IT800) equipped with Velocity Super EBSD from EDAX. A new and well-established EBSD indexing algorithm known as the spherical indexing method was employed for post-processing of results, and a detailed description is exhibited in Note S2. The XRD measurements were carried out with an X-ray diffractometer (PANalytical X′pert PRO-DY2198, Holland) with Cu Kα radiation (λ = 1.5418 Å).

### Ultrasonic imaging

An ultrasonic battery scanner (TOPS-LD50A), purchased from Wuxi Topsound Technology Co., Ltd., was used to obtain ultrasonic transmission mappings. The pouch cell was fully submerged in a silicone oil-filled test tank, and then a pair of ultrasonic focusing transducers (operating at a frequency of 2 MHz) was installed on both sides. Ultrasound waves emitted from one transducer penetrated the pouch cell, and the transmitted waves were detected by the other transducer. Subsequently, by utilizing a self-developed mapping algorithm, the peak-to-peak values of the transmitted signals were transformed into a color scale, resulting in a vibrant ultrasonic image of the internal structure.

## Supplementary Material

nwae254_Supplemental_File
