# Supplementary material for: Operando chemo-mechanical evolution in LiNi0.8Co0.1Mn0.1O2 cathodes
Source: Natl Sci Rev. 2024 Aug 5;11(9):nwae254. doi: 10.1093/nsr/nwae254 (PMC11344168; doi:10.1093/nsr/nwae254)
Supplement: nwae254_Supplemental_File [file nwae254_supplemental_file.pdf]

Supporting information

Operando chemo-mechanical evolution in $LiNi_{0.8}Co_{0.1}Mn_{0.1}O_2$ cathode

Yi Zhang[1,†], Shuaipeng Hao[1,†], Fei Pei[1], Xiangpeng Xiao[2], Chang Lu[3], Xing Lin[1], Zhe Li[4], Haijin Ji[1], Yue Shen[1], Lixia Yuan[1], Zhen Li[1,*] and Yunhui Huang[1,*]

[1]State Key Laboratory of Material Processing and Die & Mould Technology, School of Materials Science and Engineering, Huazhong University of Science and Technology, Wuhan 430074, China;

[2]School of Optical and Electronic Information, National Engineering Laboratory for Next Generation Internet Access System, Wuhan National Laboratory for Optoelectronics, Huazhong University of Science and Technology, Wuhan 430074, China;

[3]Gatan Inc. & EDAX LLC., AMETEK Commercial Enterprise (Shanghai) Co., LTD., Shanghai 200131, China;

[4]Department of physics, The Chinese University of Hong Kong, Hong Kong 999077, China

***Corresponding authors.** E-mails: li_zhen@hust.edu.cn; huangyh@hust.edu.cn

†Equally contributed to this work.

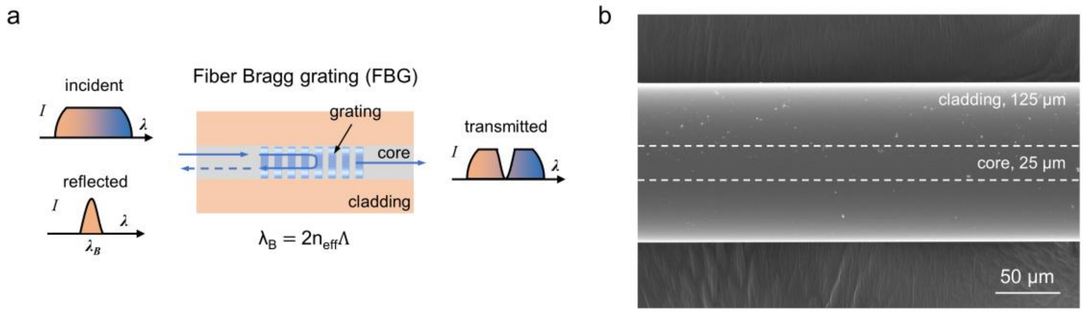

Figure S1. The work principle of FBG sensors. (a) The schematic diagram of sensing principle. (b) The top-view images of The FBG sensor.

The FBG sensor consists of cladding and core, while having a grating at a specific location. The diameter of cladding and core were 125 μm and 25 μm, respectively. The grating is inscribed on the core at any location with the length of 5 mm. When the light travels through the core, the grating can reflect the specific wavelength of light, which called Bragg wavelength and was defined as $\lambda_B = 2n_{\text{eff}}\Lambda$. Therefore, the value of Bragg wavelength depends on n_{eff} (the effective refractive index) and Λ (the grating period). Owing to the elastic-optical effect and thermos-optical effect, the changes of temperature and strain appeared in the surrounding can alter either n_{eff} or Λ, which will be visualized as the shift of Bragg wavelength.[1,2]

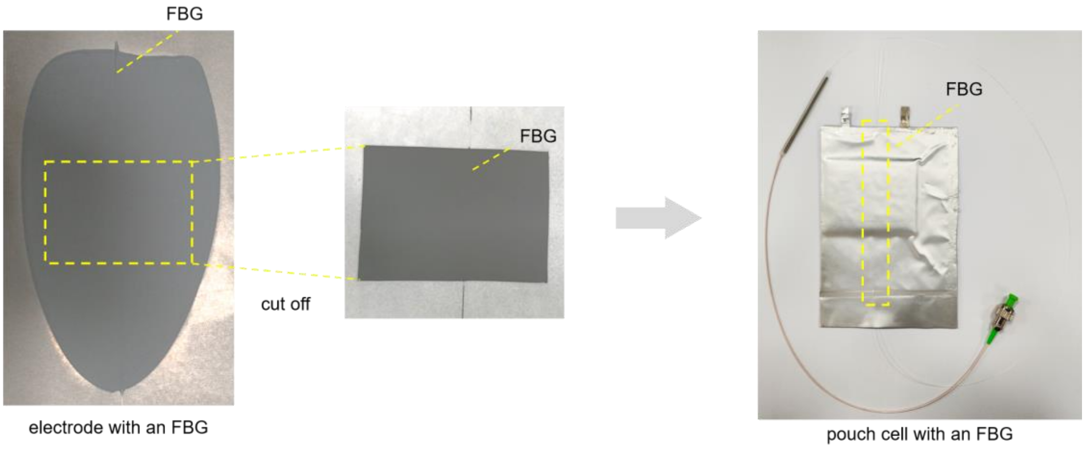

Figure S2. The digital images of the pouch cell with an implanted FBG.

According to the experimental section, the electrode with an implanted FBG was prepared. Subsequently, the electrode was cut to be appropriate size by a knife without damaging the FBG sensor. And then the electrode and lithium foil were stacked up and down, separated by two fiberglass papers. After filling with the electrolyte, the pouch cell was vacuum sealed. Ultimately, connect the FBG sensor to the jumper so that it could be connect to the optical interrogator.

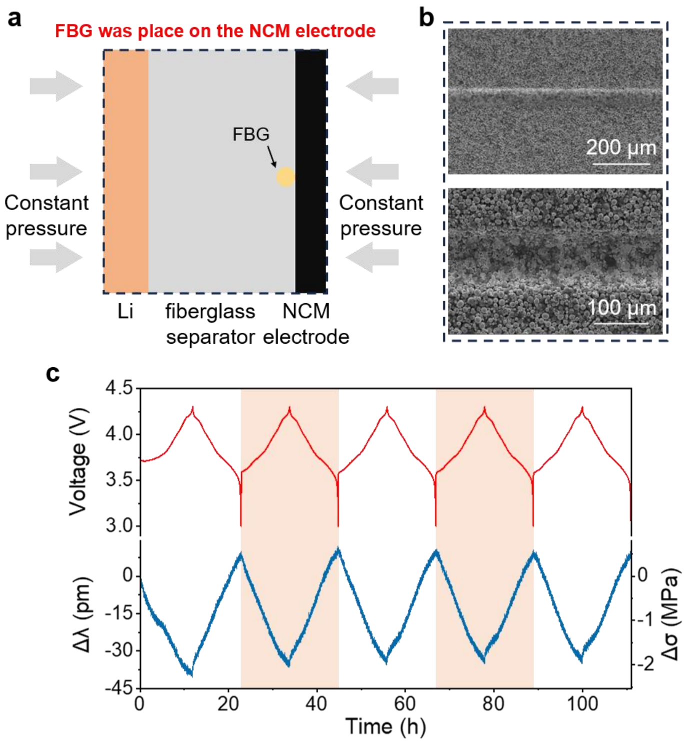

Figure S3. The stress evolution of the NCM811 electrode with an attached FBG sensor. (a) The schematic diagram of the cell assembly. The FBG sensor was placed on the surface of the NCM811 electrode. (b) The top-view images of the NCM811 electrode where the FBG sensor was. (c) Galvanostatic cycling of Li||NCM811 pouch cell, along with the stress evolution measured by the FBG sensor.

During battery assembly, the FBG sensor was placed on the surface of the electrode, the specific operation can be referred to our previous work. Due to external applied pressure, a shallow trench was formed on the electrode in the region where the FBG sensor was. According to our previous work, the thickening and thinning of the electrode caused the FBG to lengthen and shorten. Hence, the stress evolution can be seen in Figure S2c. However, the slope of the stress kept almost unchanged during charge or discharge, which cannot reflect the complex phase transition of materials.

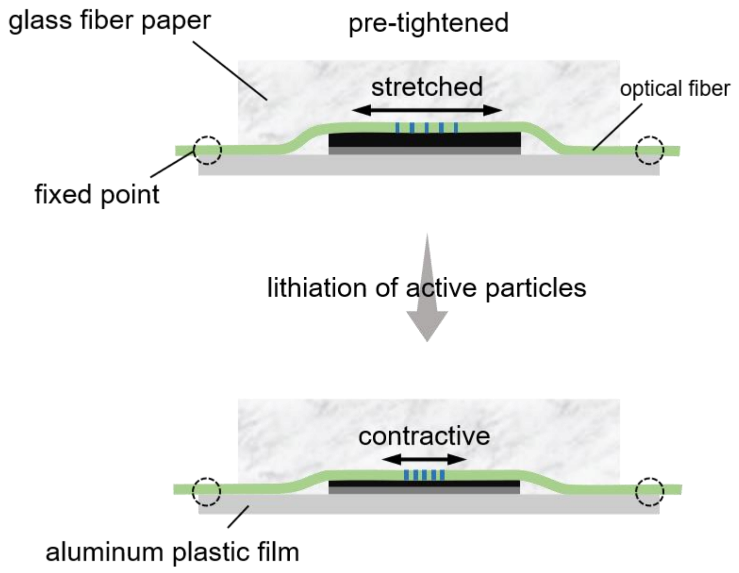

Figure S4. Schematic illustration of the attached FBG sensor and its surroundings.

During battery assembly, the FBG sensor was placed on the surface of the electrode and fixed on the aluminum plastic film by the tab film, the specific operation can be referred to our previous work.[3] During stress measurement, the constant pressure was applied on the pouch cell and both the electrode and optical fiber were pre-tightened, leading that the grating was firmly pressed onto the electrode. During delithiation of active particles, the volume reduction of active particles caused the electrode to thin, alleviating the stress state on the optical fiber. Hence, the optical fiber was stretched and shrunk repeatedly during repeated cycle, reflecting the stress evolution at electrode level.

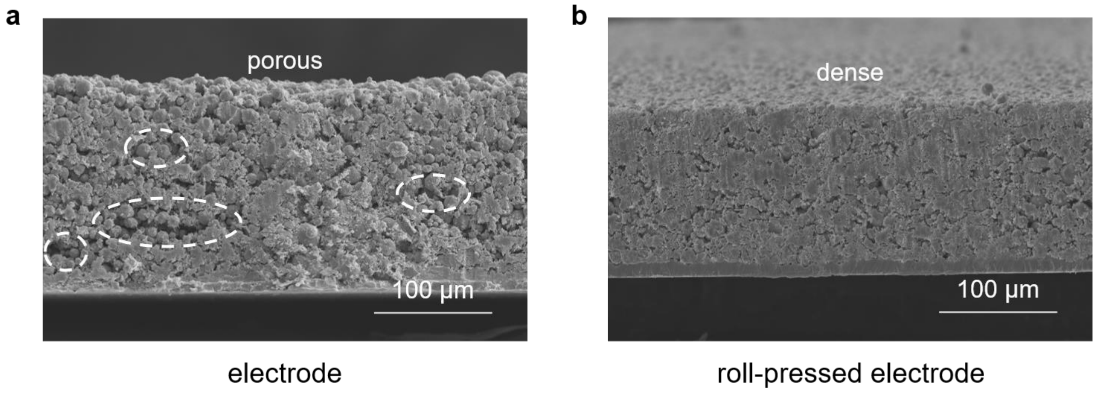

Figure S5. Cross-section images of the electrode. (a) Without being roll-pressed, the electrode was porous. (b) After being roll-pressed, the electrode became dense.

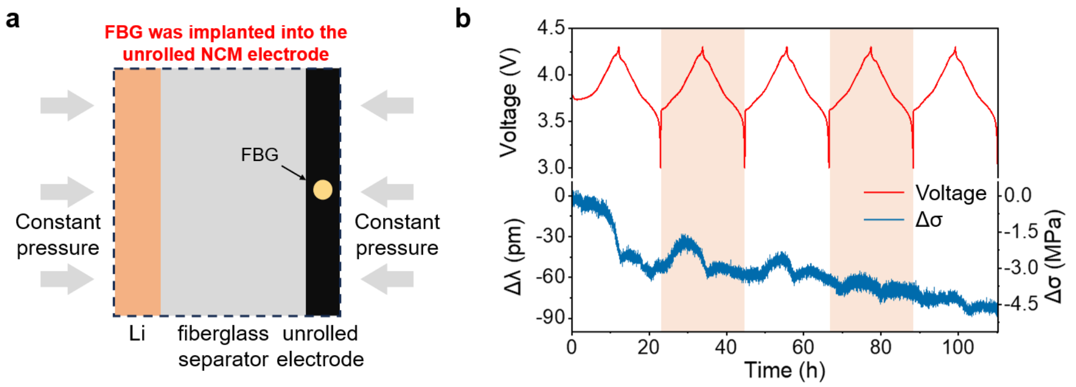

Figure S6. The stress evolution of the unroll-pressed NCM811 electrode with an implanted FBG sensor. (a) The schematic diagram of the cell assembly. The FBG sensor was implanted into the NCM811 electrode, and the electrode was not roll-pressed. (b) Galvanostatic cycling of Li||NCM811 pouch cell, along with the stress evolution measured by the FBG sensor.

Following the method of experimental section, the pouch cell was prepared to be tested. The FBG sensor was implanted into the electrode and the electrode was not roll-pressed, shown in Figure 1d and 1e. Since the electrode was not roll-pressed, the holes could balance the volume changes of the NCM materials. Therefore, the stress changes were not periodic and remained basically constant. This configuration made it difficult to monitor the stress evolution.

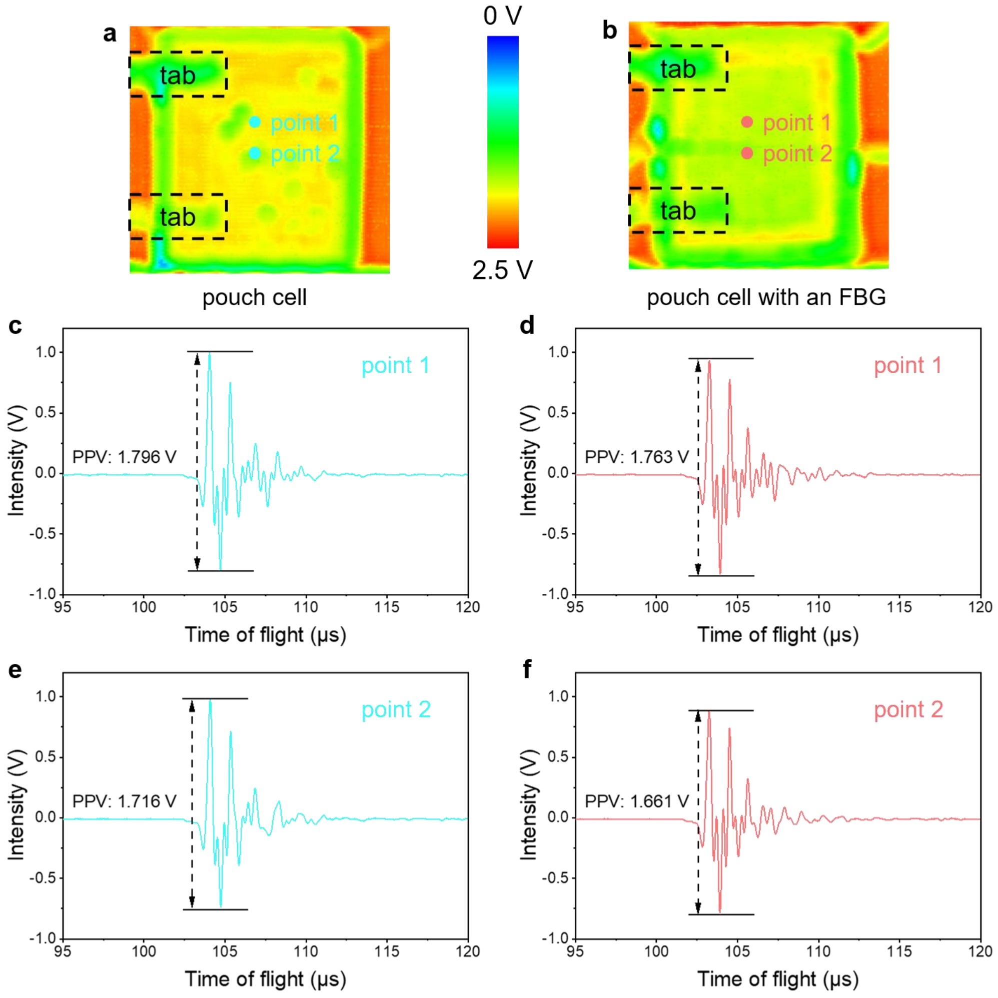

Figure S7. The ultrasonic transmission images of the pouch cell and the corresponding ultrasonic waves. (a, c, e) The ultrasonic transmission images of the pouch cell without an FBG (a) and the corresponding ultrasonic waves of two points (c, e). (b, d, f) The ultrasonic transmission images of the pouch cell with an FBG (b) and the corresponding ultrasonic waves of two points (d, f). Therein, point 2 is located at the FBG sensor.

According to the ultrasonic transmission images, the FBG sensor had little effect on the wettability of the pouch cell. As shown in Figure S6c and S6e, the peak-to-peak value are equal to 1.7-1.8 V. As for the pouch cell with an FBG sensor, the peak-to-peak value of the FBG sensor is slightly lower (point 2) than other locations (point 1). Such a small difference does not have any effect on battery performance.

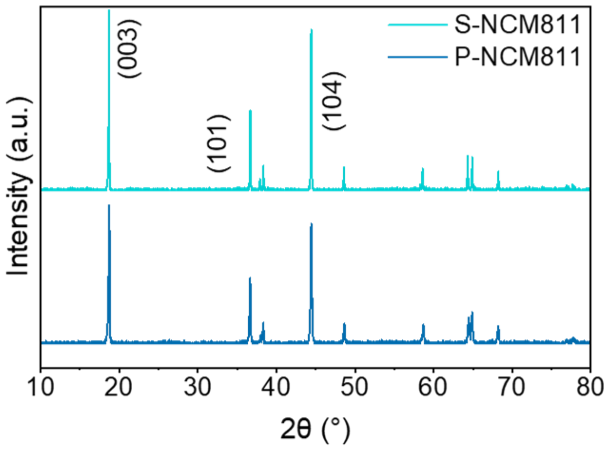

Figure S8. XRD results of P-NCM811 and S-NCM811.

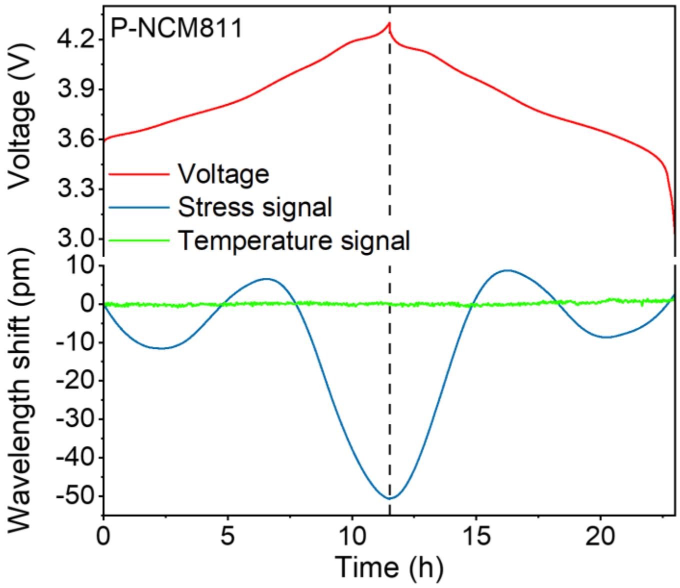

Figure S9. The measured wavelength shift of these two FBG sensors. The first FBG sensor was implanted into the electrode, which was used to obtain the stress information. Another FBG sensor was loosely placed on the pouch cell, which was used to measure temperature.

Due to the strictly controlled temperature, it can be found that the temperature barely changed and only a slight flutter occurred. Removing the interference of temperature, the stress evolution can be obtained (Figure 2c). All the subsequent stress evolution is obtained in this way, and the temperature signal is not be shown later.

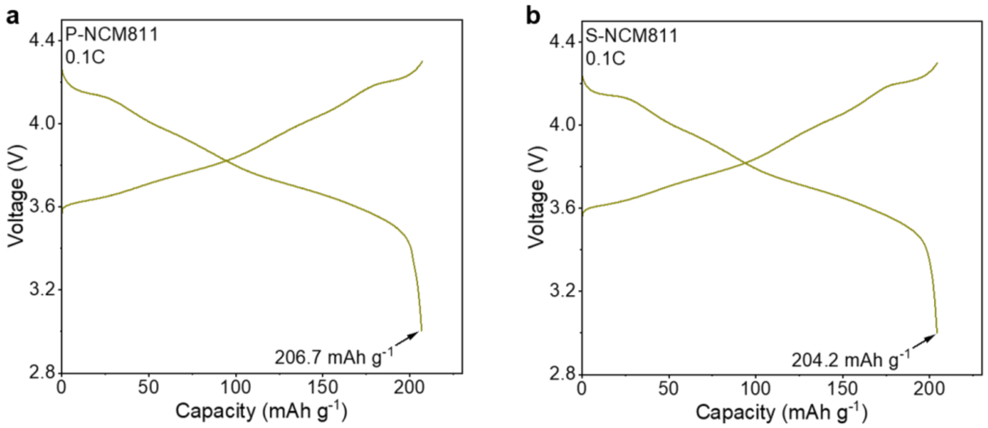

Figure S10. Voltage profiles of P-NCM811 (a) and S-NCM811 (b) at 0.1C.

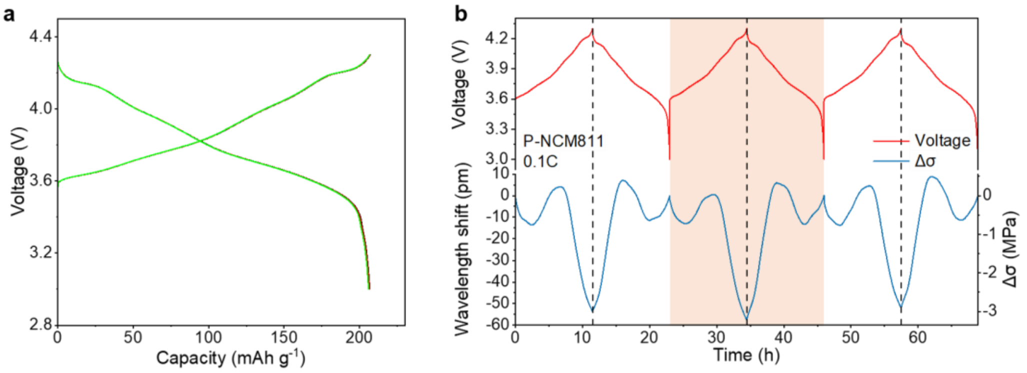

Figure S11. Stress evolution of P-NCM811 materials at 0.1C. (a) Corresponding capacity profile of P-NCM811 materials at 0.1C for 3 cycles. (b) The stress evolution of P-NCM materials, with the corresponding voltage curves for 3 cycles.

At a rate of 0.1C, the capacity of P-NCM811 materials is almost not reduced, indicating the good chemical reversibility. Accordingly, the stress evolution also had periodicity and swung with voltage. Meanwhile, the trend of stress evolution is similar of each cycle, exhibiting the good mechanical reversibility.

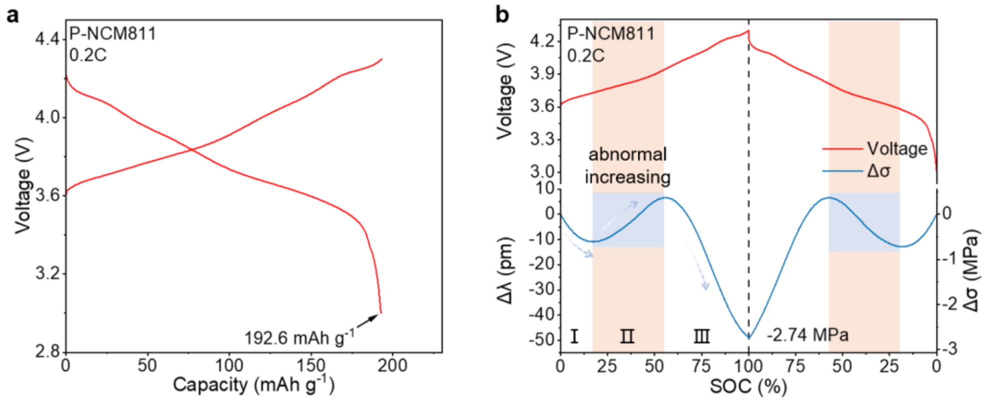

Figure S12. Stress evolution of P-NCM811 materials at 0.2C. (a) Corresponding capacity profile of P-NCM811 materials at 0.2C. (b) The stress evolution of P-NCM materials, with the corresponding voltage curves.

At a higher rate of 0.2C, P-NCM811 materials delivered lower capacity of 192.6 mAh g^{-1}. Accordingly, due to capacity-dependent chemical strain, the minimum value of $\Delta\sigma$ was also reduced. However, the trend of stress evolution has not changed and the abnormal increasing of stress during charge still exist.

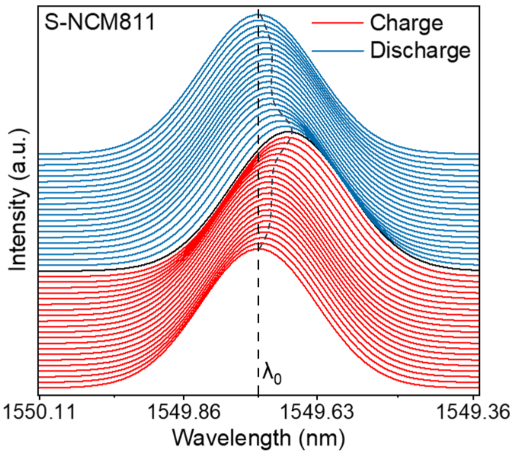

Figure S13. 2D stack-view of the reflected spectra given by the FBG sensor implanted into the S-NCM811 electrode during cycling.

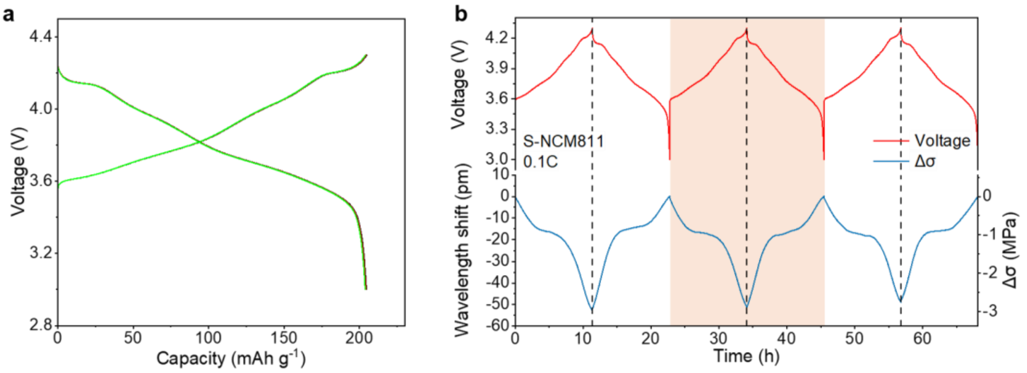

Figure S14. Stress evolution of S-NCM811 materials at 0.1C. (a) Corresponding capacity profile of S-NCM811 materials at 0.1C for 3 cycles. (b) The stress evolution of S-NCM materials, with the corresponding voltage curves for 3 cycles.

At a rate of 0.1C, the capacity of S-NCM811 materials is almost not reduced, indicating the good chemical reversibility. Accordingly, the stress evolution also had periodicity and swung with voltage. Meanwhile, the trend of stress evolution is similar of each cycle, exhibiting the good mechanical reversibility.

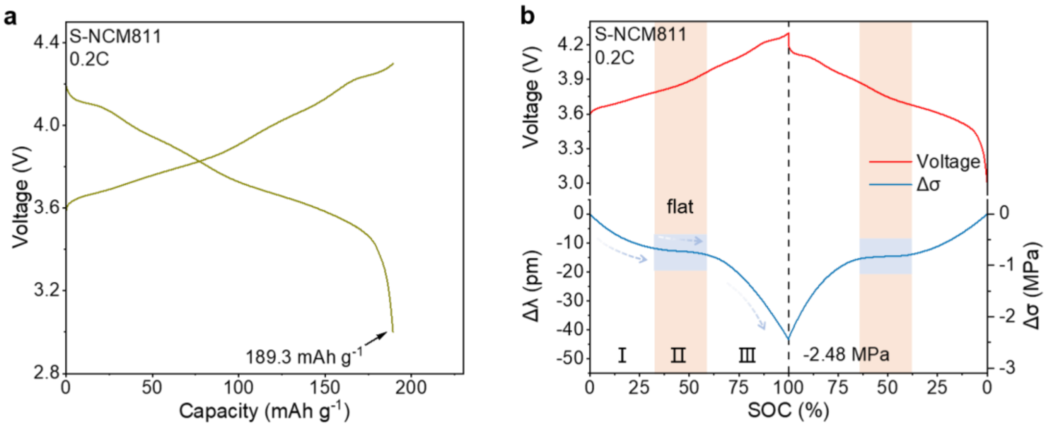

Figure S15. Stress evolution of S-NCM811 materials at 0.2C. (a) Corresponding capacity profile of S-NCM811 materials at 0.2C. (b) The stress evolution of S-NCM811 materials, with the corresponding voltage curves.

At a higher rate of 0.2C, S-NCM811 materials delivered lower capacity of 189.3 mAh g^{-1}. Similar to P-NCM811 materials, except for the reduced minimum value of $\Delta\sigma$, the trend of stress evolution has not changed.

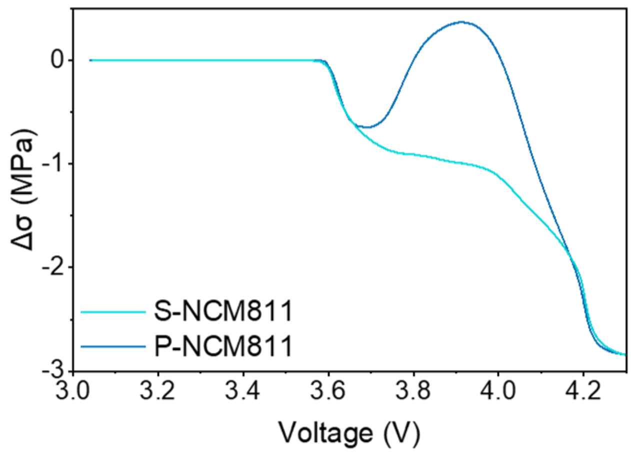

Figure S16. Voltage-resolved Δσ profile of S-NCM811 and P-NCM811 materials.

Since Δσ and voltage are both time dependent, Δσ is taken as a function of voltage. Due to the effect of polarization, Δσ remained unchanged at voltage less than 3.6 V, meaning that no chemical reactions took place. When they were charged to 3.7V, the Δσ of these two materials began to differ, which was called stress anomaly interval.

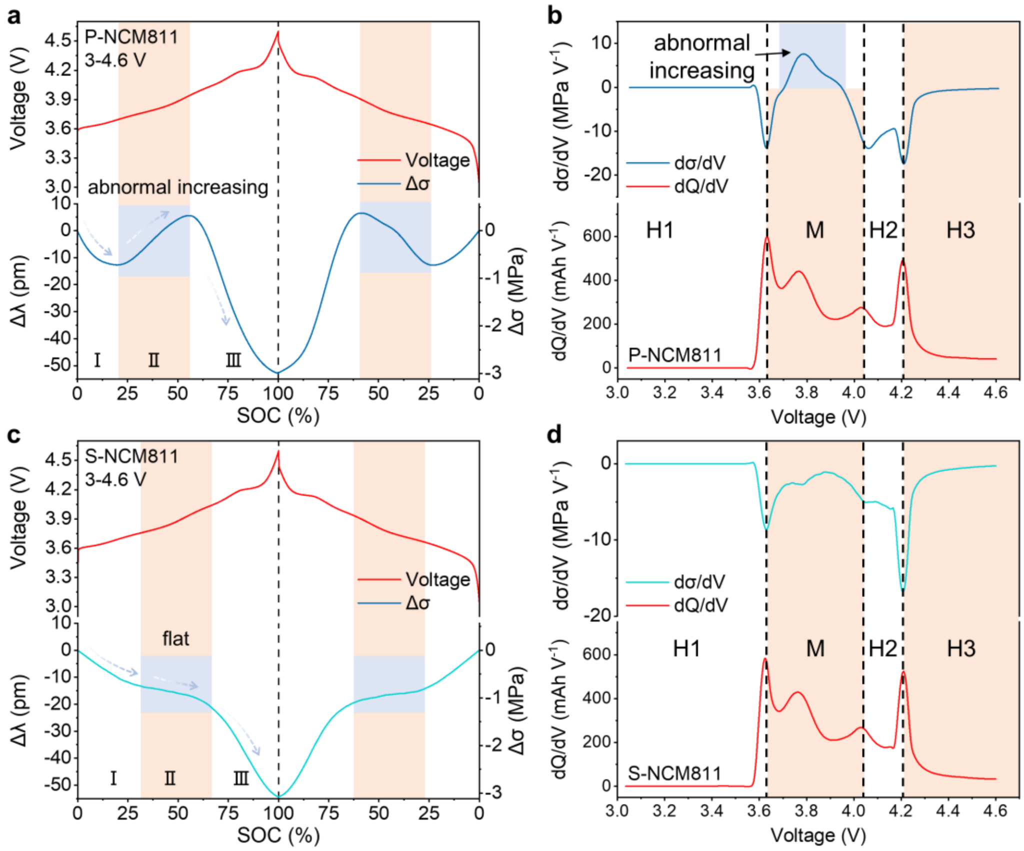

Figure S17. The stress evolution at the high voltage of 3-4.6 V. (a) The stress evolution of P-NCM811 materials, with the corresponding voltage curves. (b) Voltage-resolved dσ/dV profile together with the dQ/dV plot of P-NCM811 materials. (c) The stress evolution of S-NCM811 materials, with the corresponding voltage curves. (d) Voltage-resolved dσ/dV profile together with the dQ/dV plot at the high voltage of S-NCM811 materials.

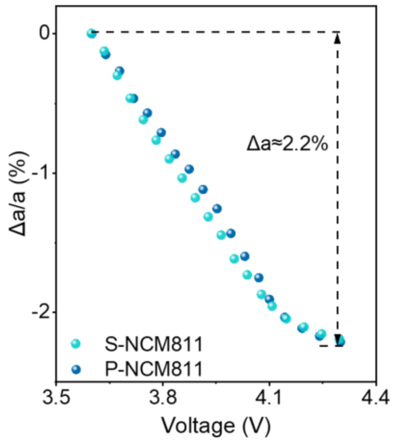

Figure S18. The variation of a-axis parameter for P-NCM811 and S-NCM811 during charging process.

The variation of a-axis parameter for P-NCM811 and S-NCM811 is almost at the same, with the variation of 2.2%.

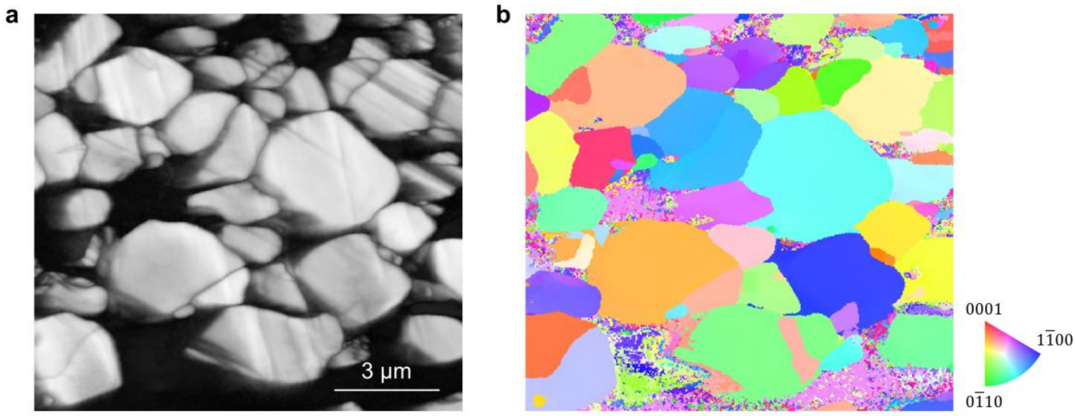

Figure S19. EBSD results of S-NCM811. (a) SEM images of cross-sections of S-NCM811 particles. (b) EBSD images and crystal plane distribution of S-NCM811 particles.

Obviously, single crystal particles are distinct from each other. The black region in Figure S19a represents the absence of single crystal particles, appeared as multicolored blobs in Figure S19b. Each single crystal particle exhibited different color in Figure S19b, indicating the different orientation of each single crystal particles. However, each particle is independent and there is no strong interaction between each particle, which leaded to no cracks and facilitated the monotonicity of stress evolution during charge/discharge

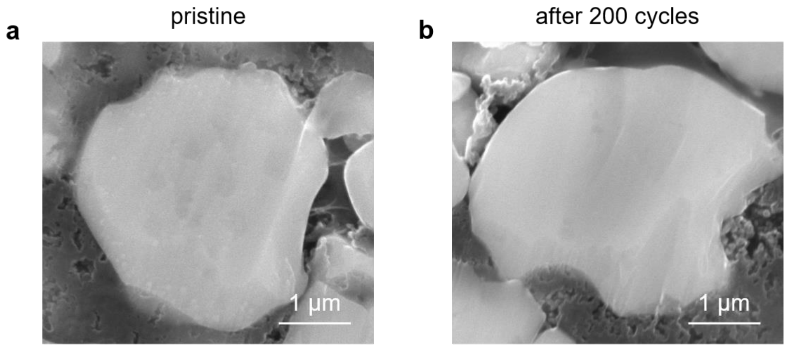

Figure S20. Cross-section of S-NCM811 particles before cycling (a) and after 200 cycles (b).

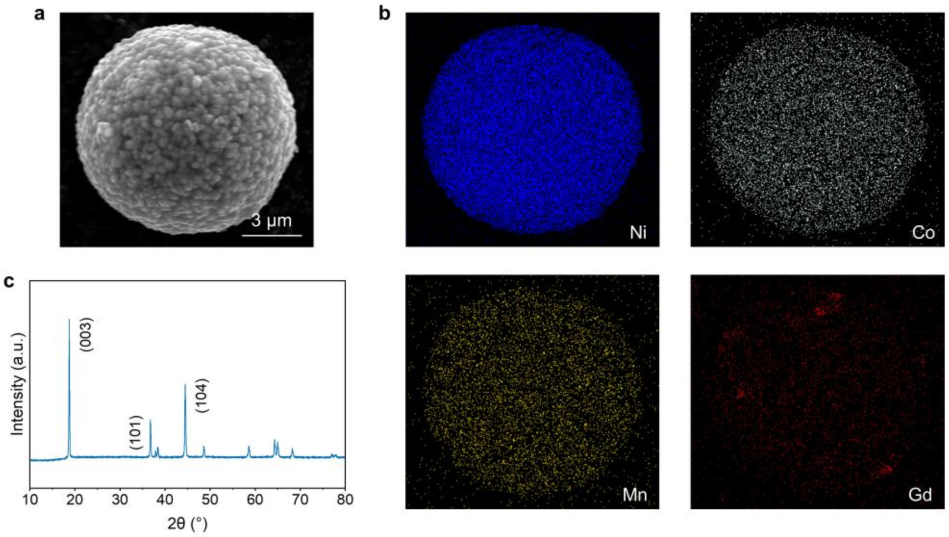

Figure S21. The characterization of OAS-NCM811 materials. (a-b) The scanning electron images of Gd-doped P-NCM811 materials with the corresponding elemental mapping of Ni, Co Mn and Gd. (c) XRD results of OAS-NCM811.

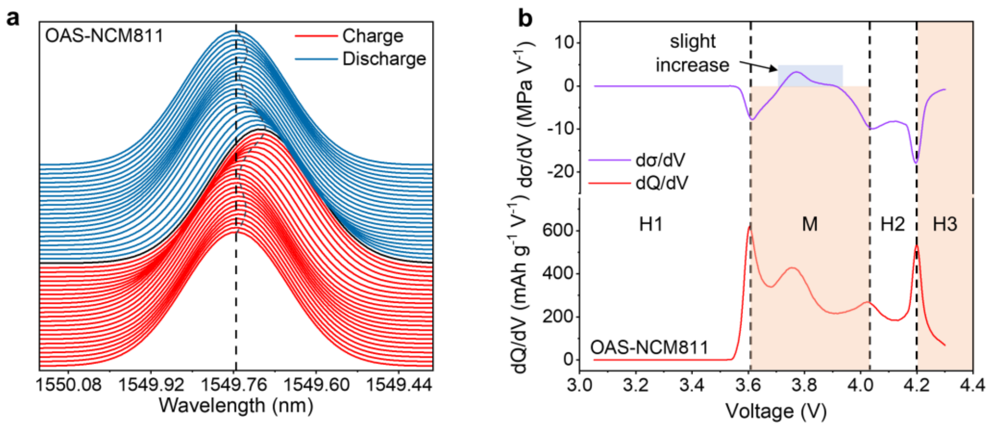

Figure S22. The related results of stress evolution of OAS-NCM811 materials. (a) 2D stack-view of the reflected spectra given by the FBG sensor implanted into the OAS-NCM811 electrode during cycling. (b) Voltage-resolved dσ/dV profile together with the dQ/dV plot of OAS-NCM811 materials.

As for OAS-NCM811 materials, the dσ/dV plot also exhibits a peak near the voltage of phase transition, indicating that phase transition leaded a sudden change in stress. Especially, due to the decrease of structural stress, the value of the plot in blue region decreased significantly.

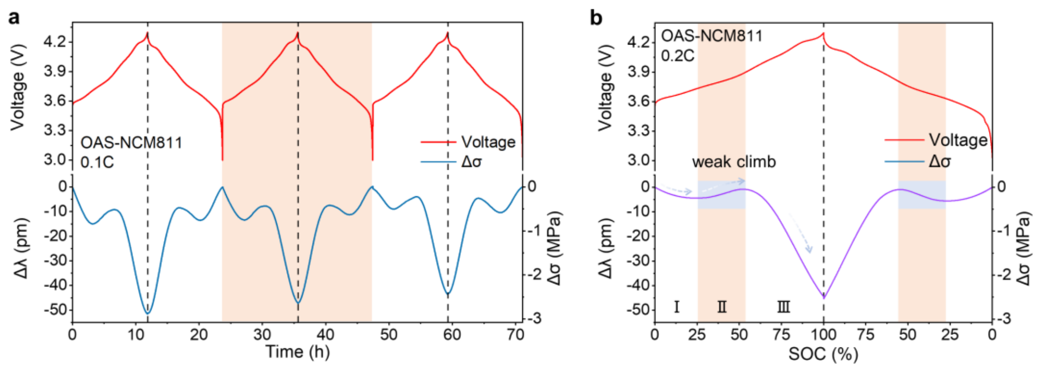

Figure S23. The related results of stress evolution of OAS-NCM811 materials. (a) The stress evolution of OAS-NCM materials, with the corresponding voltage curves for 3 cycles. (b) The stress evolution of OAS-NCM811 materials at 0.2C, with the corresponding voltage curves.

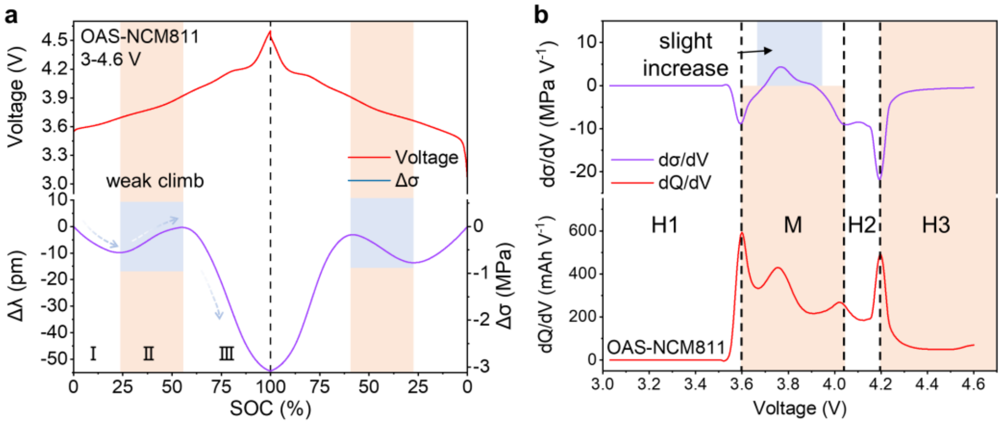

Figure S24. The stress evolution at the high voltage of 3-4.6 V for OAS-NCM811 materials. (a) The stress evolution with the corresponding voltage curves. (b) Voltage-resolved dσ/dV profile together with the dQ/dV plot.

Similarly, the wide voltage has not influenced the trend of stress evolution and caused a slight increase in stress due to the increased chemical stress.

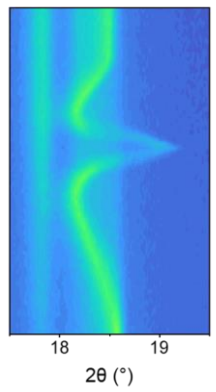

Figure S25. The in-situ XRD characterization of OAS-NCM811 materials.

Although the peak shift of (003) still increased and then decreases, the amplitude of changes was only 0.89 °, which is smaller than that of P-NCM811 materials. Hence, the variation of c-axis parameter can be smaller, resulting in smaller changes of crystal shape.

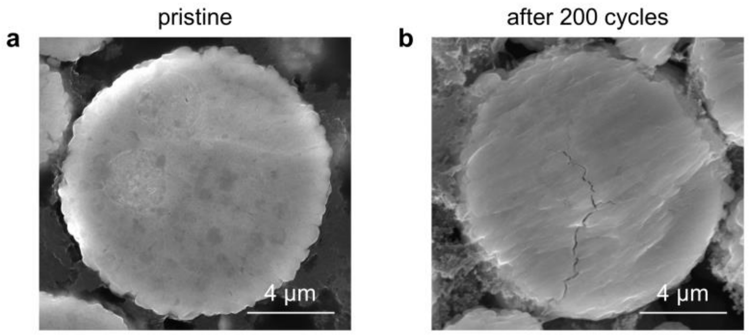

Figure S26. Cross-section of OAS-NCM811 particles before cycling (a) and after 200 cycles (b).

Supplementary Note 1 | The calculation of the transformation of $\Delta\lambda_B$ (pm) into stress (MPa)

According to the principle of the FBG sensor for stress measurement, when the FBG is stretched, the shift of λ_B can be defined as:

$$\frac{\Delta\lambda_B}{\lambda_{B,0}} = (1 - p_e) = \left(1 - \frac{n_{\text{eff}}^2[p_{12} - \nu(p_{11} + p_{12})]}{2}\right)\varepsilon \qquad \text{(Equation 1)}$$

Where p_{11} and p_{12} represent the strain-optical coefficients, ν is the Poisson's ratio. For silica materials, these material property values are all constant and known ($n_{\text{eff}} = 1.45$, $p_{11} = 0.113$, $p_{12} = 0.252$, $\nu = 0.17$). Then the equation 1 can be replaced by:

$$\frac{\Delta\lambda_B}{\lambda_{B,0}} = \left(1 - \frac{n_{\text{eff}}^2[p_{12} - \nu(p_{11} + p_{12})]}{2}\right)\varepsilon = C\varepsilon \qquad \text{(Equation 2)}$$

Where C can be calculated with a constant value of 0.8003150625. Hence, ε can be expressed as:

$$\varepsilon = \frac{\Delta\lambda_B}{C\lambda_{B,0}} \qquad \text{(Equation 3)}$$

Furthermore, stress (σ) can be obtained through Hooke's law:

$$\sigma = E\varepsilon = \frac{E}{C\lambda_{B,0}}\Delta\lambda_B = k_\sigma\Delta\lambda_B \qquad \text{(Equation 4)}$$

where E is Young's modulus of silica optical fiber sensors (69.9 GPa). Therefore, the stress is related to initial Bragg wavelength and its shift during measurement. In our work, the Bragg wavelength at the initial time was 1535-1550 nm universally. Plug these known values into equation 4, the coefficient k_σ can be obtained as below:

Table S1 The coefficient k_σ corresponds to the initial Bragg wavelength.

Initial Bragg wavelength (nm)	1535	1540	1545	1550
k_σ (MPa pm^{-1})	0.056899	0.056715	0.056531	0.056349

It was not hard to find that k_σ changed a little when initial Bragg wavelength varied much. Therefore, initial Bragg wavelength was considered as a constant (1540 nm) for convenient and k_σ was equal to 0.056715 MPa pm^{-1}.

Supplementary Note 2 | The post processing of EBSD results

The raw EBSD patterns of these samples were somewhat blurred due to the sample's sensitivity to the electron beam, making traditional Hough indexing ineffective for accurate band detection. To address this, we employed a new and well-established EBSD indexing algorithm known as the spherical indexing method. [4-7]

Initially, a forward-based physics model was applied to simulate electron interactions with the sample under the same EBSD measurement geometry on the JEOL microscope. In the EDAX OIM 9 software, 2 billion electrons with a primary energy of 20 kV were set to strike the sample's crystal structure to produce a Master Pattern (Kikuchi Sphere), containing all its Kikuchi patterns in real space. The experimentally acquired Kikuchi patterns were then back-projected onto this Master Pattern. By applying a spherical harmonic transform, the experimental pattern was cross-correlated with the spherical image from the Master Pattern. The best fit for each pattern was determined by the highest spherical confidence index (CI) value. Data points with a spherical CI value less than 0.1 were removed from the final image due to low accuracy.

REFERENCE

1. Albero Blanquer L, Marchini F, Seitz JR *et al.* Optical sensors for *operando* stress monitoring in lithium-based batteries containing solid-state or liquid electrolytes. *Nat Commun* 2022; **13**: 1153.
2. Li Y, Zhang Y, Li Z *et al. Operando* decoding of surface strain in anode-free lithium metal batteries via optical fiber sensor. *Adv Sci* 2022; **9**: 2203247.
3. Zhang Y, Xiao X, Chen W *et al. In operando* monitoring the stress evolution of silicon anode electrodes during battery operation via optical fiber sensors. *Small* 2024: 2311299; doi: 10.1002/smll.202311299
4. Lenthe WC, Germain L, Chini MR *et al.* Spherical indexing of overlap EBSD patterns for orientation-related phases – Application to titanium. *Acta Mater* 2020; **188**: 579-90.
5. Jackson MA, Pascal E, De Graef M. Dictionary indexing of electron back-scatter diffraction patterns: a hands-on tutorial. *Integr Mater Manuf I* 2019; **8**: 226-46.
6. Hielscher R, Bartel F, Britton TB. Gazing at crystal balls: Electron backscatter diffraction pattern analysis and cross correlation on the sphere. *Ultramicroscopy* 2019; **207**: 112836.
7. Lenthe WC, Singh S, Graef MD. A spherical harmonic transform approach to the indexing of electron back-scattered diffraction patterns. *Ultramicroscopy* 2019; **207**: 112841.
